# Dihydroorotate dehydrogenase (DHODH) regulates trophoblast syncytialization through organelle stress–induced cellular senescence

**DOI:** 10.1002/2211-5463.70194

**Published:** 2026-01-16

**Authors:** Kanoko Yoshida, Kazuya Kusama, Ayaka Horiuchi, Yu Kawaguchi, Atsuya Tsuru, Mikihiro Yoshie, Kazuhiro Tamura

**Affiliations:** ^1^ Laboratory of Endocrine Pharmacology Tokyo University of Pharmacy and Life Sciences Japan

**Keywords:** cellular senescence, dihydroorotate dehydrogenase, human trophoblast stem cells, Hypertensive disorder of pregnancy, mitochondrial dysfunction, syncytialization

## Abstract

Trophoblast syncytialization is essential for placental function, and its dysregulation contributes to hypertensive disorders of pregnancy (HDPs), which compromise maternal and fetal health. Reduced expression of mitochondrial dihydroorotate dehydrogenase (DHODH) was observed in early‐onset HDP placentas in our previous study. Experiments using human trophoblast stem cells demonstrated that DHODH inhibition impairs syncytialization and induces cellular senescence via mitochondrial and endoplasmic reticulum stress, elevating sFlt1/PlGF levels, a hallmark of placental dysfunction in HDPs. Mitochondrial activators quercetin and riboflavin partially reversed these effects. Our findings suggest that DHODH may be a key regulator of trophoblast differentiation by linking organelle stress to cellular senescence.

## Graphical abstract

The inhibition of mitochondrial dihydroorotate dehydrogenase (DHODH) impairs syncytialization and induces cellular senescence via mitochondrial and endoplasmic reticulum stress in human trophoblast stem cells, elevating sFlt1/PlGF levels, a hallmark of placental dysfunction in hypertensive disorders of pregnancy.

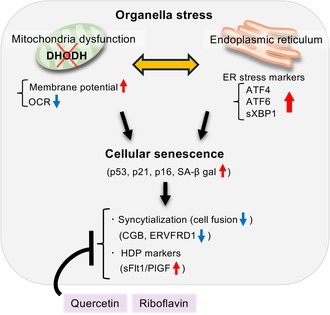

**Abbreviations**
DHODH: dihydroorotate dehydrogenase
ER: endoplasmic reticulum
EVTs: extravillous trophoblasts
hCG: human chorionic gonadotropin
HDPs: hypertensive disorder of pregnancy
PlGF: placental growth factor
ROS: reactive oxygen species
sFlt1: soluble fms‐like tyrosine kinase‐1
STs: syncytiotrophoblasts
TSCs: human trophoblast stem cells
VEGF: vascular endothelial growth factor

The placenta is an essential organ that supports pregnancy and fetal growth. It is primarily composed of trophoblast cells, which are derived from trophoblast stem cells (TSCs). TSCs possess the capacity for long‐term self‐renewal and can differentiate into all trophoblast cell types that constitute the placenta. Of these cell types, cytotrophoblasts differentiate and fuse into multinucleated syncytiotrophoblasts (STs), a process known as syncytialization, which establishes the maternal–fetal interface, mediates nutrient and gas exchange, and provides a protective barrier against viral infections [1, 2]. In addition, STs secrete key hormones such as human chorionic gonadotropin (hCG), essential for maintaining pregnancy, and placental lactogen, which regulates maternal metabolism and supports mammary gland development [3]. Impaired syncytialization results in failure of placentation, which may lead to pregnancy complications such as hypertensive disorders of pregnancy (HDPs) [4].

HDPs are characterized by the new onset of hypertension and proteinuria after 20 weeks of gestation, affecting approximately 10% of pregnant women. HDPs constitute a serious obstetric complication that poses substantial risks to both the mother and fetus, potentially progressing to severe outcomes such as eclampsia and maternal multi‐organ dysfunction [5]. Maternal chronic hypertension, pre‐existing diabetes, and advanced maternal age (> 40 years) are recognized as major risk factors for HDPs [6, 7]. Clinically, placentas from patients with HDPs show elevated levels of the angiogenic factor soluble fms‐like tyrosine kinase‐1 (sFlt1), a receptor for vascular endothelial growth factor (VEGF), and reduced levels of placental growth factor (PlGF) [8].

Organelle stress, including mitochondrial dysfunction and endoplasmic reticulum (ER) stress, has been implicated in placental dysfunction in HDPs. Previously, we reported that mitochondrial dysfunction in STs leads to excessive accumulation of reactive oxygen species, which suppressed syncytialization, and that a mitochondrial activator quercetin restored these impairments [9]. In normal pregnancies, intracellular stress in trophoblasts increases with advancing gestational age, suggesting that an appropriate level of stress constitutes a physiological characteristic of the placenta; however, excessive and chronic stress may promote trophoblast senescence [10]. These findings indicate that organelle stress and cellular senescence are closely interconnected, and such mechanisms may also contribute to the pathophysiology of HDPs.

In our RNA‐seq analysis, we previously demonstrated that the expression of dihydroorotate dehydrogenase (DHODH) is reduced in placental tissues from HDP patients [11]. DHODH is a mitochondrial oxidoreductase that plays a crucial role in the *de novo* biosynthesis of pyrimidine nucleotides [12]. In addition to pyrimidine nucleotide biosynthesis, since DHODH is localized in the mitochondrial inner membrane, it is functionally linked to mitochondrial energy metabolism via the electron transport chain [13].

However, the relationship between DHODH and organelle stress, as well as its impact on placentation, has not yet been investigated. In this study, we evaluated the effects of DHODH inhibition on organelle stress, cellular senescence, syncytialization, and HDP severity markers using TSCs.

## Materials and methods

### Culture of human trophoblast stem cells

TSCs (RIKEN BRC, CT27, Ibaraki, Japan) were cultured on Laminin‐511 (1 : 2000, iMatrix‐511 silk, Matrixome, Osaka, Japan)‐coated plates in the basal medium composed of 1 : 1 Ham's F12/Dulbecco's modified Eagle's medium (DMEM/F12) supplemented with 0.3% BSA (Fujifilm Wako Pure Chemical Corp., Osaka, Japan), 1% ITS‐X (Fujifilm Wako Pure Chemical Corp), 1% Penicillin–Streptomycin (Thermo Fisher Scientific, Waltham, MA, USA), 0.1 mm 2‐mercaptoethanol (Thermo Fisher Scientific), 50 ng·mL^−1^ EGF (Fujifilm Wako Pure Chemical Corp), 0.2% fetal bovine serum (FBS, Nichirei Biosciences), and 1.5 μg·mL^−1^ L‐ascorbic acid (Tokyo Chemical Industry, Tokyo, Japan). The complete trophoblast stem (TS) cell medium was prepared by supplementing 5 μm Y27632, 160 μm valproic acid, and an inhibitor cocktail (2 μm CHIR99021, 0.5 μm A83‐01, and 1 μm SB431542). All reagents were obtained from Fujifilm Wako Pure Chemical Corp. [14].

For syncytiotrophoblast differentiation, TSCs were seeded at 7 × 10^4^ cells per well in 12‐well plates and cultured for 48 h in the differentiation medium based on the basal medium formulation but modified by replacing the TS supplements with 2.5 μm Y27632, 2.5 μm forskolin (FSK, Cayman chemical, Ann Arbor, MI USA), and 4% KnockOut Serum Replacement (Thermo Fisher Scientific) without valproic acid, L‐ascorbic acid, and FBS, and cells were harvested for analysis.

Cells were treated with the following compounds: uridine (Uri, 100 μm, Sigma‐Aldrich, Tokyo, Japan), DHODH inhibitors [orludodstat; Orlu, 1 nm (Selleck Chemicals, TX, USA), brequinar; Bre, 25 nm (Selleck Chemicals)]; mitochondrial complex inhibitors [rotenone; Rote, 50 nm (Fujifilm Wako Pure Chemical Corp), 2‐thenoyltrifluoroacetone; TTFA, 500 μm (Sigma‐Aldrich), antimycin A; Anti, 50 nm (Fujifilm Wako Pure Chemical Corp), and potassium cyanide; KCN, 500 μm (Fujifilm Wako Pure Chemical Corp)]; ER stress inducers [tunicamycin; Tuni, 1 μm (Fujifilm Wako Pure Chemical Corp) and thapsigargin; Thap, 50 nm (Fujifilm Wako Pure Chemical Corp)]; and mitochondrial activators [quercetin; Que, 5 μm (Tokyo Chemical Industry, Tokyo, Japan) and riboflavin; Ribo, 25 μm (Sigma‐Aldrich)].

### RNA extraction and quantitative RT‐PCR

RNA was extracted using the RNeasy Mini Kit (Qiagen, Tokyo, Japan), according to the manufacturer's instructions. Reverse transcription of mRNA was performed using a ReverTra Ace qPCR RT Kit (Toyobo, Osaka, Japan), and the cDNA produced was subjected to qPCR amplification in a THUNDERBIRD Next SYBR qPCR Mix (Toyobo). The primers used are listed in Table S1. Calibration curves were used to determine the amplification of each target gene with respect to the expression of a reference gene, glyceraldehyde‐3‐phosphate dehydrogenase (GAPDH). The mean crossing threshold (*C*_t_) values for each target were calculated using the Sequence Detection System software v2.3 (Thermo Fisher Scientific) [15].

### Cell fusion assay

TSCs were fixed with methanol and incubated with anti‐E‐cadherin antibody (1 : 200, #3195, Cell Signaling Technologies, Tokyo, Japan) and AlexaFluor 594‐conjugated goat anti‐mouse antibody (Thermo Fisher Scientific) to distinguish cell surfaces. The nuclei were counterstained with 4′,6‐diamino‐2‐phenylindole 2HCl (DAPI). Images were collected using a microscope (BZ‐X810, Keyence, Osaka, Japan). The number of nuclei in syncytiotrophoblasts and the total number of nuclei were counted in five randomly selected microscopic areas per sample, and the fusion index was calculated [(number of nuclei in syncytia/total number of nuclei) × 100] [9]. Counting was performed by an investigator blinded to the experimental conditions. The data are presented as ratios relative to the control and shown as mean ± SEM from three independent experiments.

### DHODH activity assay

TSCs were detached using trypsin, washed with phosphate‐buffered saline (PBS) for preparation of cell lysates, cells were subjected to sonication in distilled water for 10 min on ice, followed by centrifugation at 16 000 ***g*** for 20 min to remove debris. The resulting supernatant was used as the enzyme source for the DHODH activity assay. For the enzyme reaction, cell lysates were incubated in 200 μL of reaction buffer containing 500 μm dihydroorotic acid (Sigma‐Aldrich), 100 μm coenzyme Q_10_ (Tokyo Chemical Industry), 0.2% Triton X‐100 (Fujifilm Wako Pure Chemical Corp), and 200 mm K_2_CO_3_–HCl buffer (pH 8.0) at 37 °C for 1 h. After incubation, 100 μL of the reaction mixture was combined with 4.0 mm 4‐trifluoromethylbenzamidoxime (Sigma‐Aldrich), 8.0 mm K_3_ [Fe (CN)_6_] (Fujifilm Wako Pure Chemical Corp), and 80 mm K_2_CO_3_ buffer (Fujifilm Wako Pure Chemical Corp), and the mixture was heated at 80 °C for 4 min to generate a fluorescent product derived from orotic acid. The fluorescence intensity was measured at an excitation wavelength of 340 nm and an emission wavelength of 460 nm using a microplate reader [16]. DHODH activity was relative activity compared with the untreated control.

### RNA‐sequencing (RNA‐Seq), gene ontology (GO), and pathway analyses

For RNA‐seq analysis, RNA was extracted using a RNeasy Mini Kit (Qiagen) according to the manufacturer's instructions. High‐throughput sequencing libraries were prepared using a TruSeq Stranded Total RNA LT Sample Prep Kit (Illumina, San Diego, CA, USA) according to the manufacturer's instructions, and data analysis was performed by Macrogen Japan (Kyoto, Japan). Primary sequence data were deposited in the DDBJ (DNA Data Bank of Japan) Sequence Read Archive (https://www.ddbj.nig.ac.jp/dra/index‐e.html; accession number: DRA021721). Data analysis was performed as described previously [17]. Briefly, trimmed sequences were analyzed using the STAR/RSEM/edgeR pipeline, the human genome (hg38), and reference annotations obtained from the UCSC genome browser (https://genome.ucsc.edu). Significantly, differentially expressed genes (DEGs) were identified on the basis of counts per million (CPM) levels. GO and enriched signaling pathway analyses were performed using the Enrichr tool (http://amp.pharm.mssm.edu/Enrichr/).

### Senescence‐associated beta‐galactosidase staining

Cells were plated on 18‐well (ibidi, Fitchburg, WI, USA), and SPiDER‐β‐Gal working solution (Dojindo, Kumamoto, Japan) was added to the plate for 30 min at 37 °C. The cells were mounted on microscope slides in mounting medium containing DAPI to label the nuclei. Senescent cells were counted in five randomly selected microscopic fields per sample across three independent experiments. Images were collected using a microscope (BZ‐X810, Keyence).

### Mitochondrial membrane potential assay

Cells were seeded into 24‐well coated with Matrigel (Corning, Corning NY, NY, USA) and cultured at 37 °C for 48 h. The mitochondrial membrane potential was evaluated using the MT‐1 MitoMP Detection Kit (Dojindo) following the manufacturer's protocol. Cells incubated with MT‐1 working solution for 30 min at 37 °C were detected using a BZ‐X810 fluorescence microscope (Keyence). The fluorescence was measured using a microcell count system (Keyence). Relative staining intensity was calculated (fluorescence intensity of mitochondrial membrane potential/total staining area) [9]. The data are presented as ratios of the control and shown as mean ± SEM from three independent experiments.

### Oxygen consumption rate (OCR) assay

OCR was measured using the Extracellular Oxygen Consumption Assay Kit (Dojindo) according to the manufacturer's instructions. TSCs were seeded in 96‐well plates and cultured for 24 h. The culture medium was then replaced with assay buffer containing the OCR probe together with DHODH inhibitors, mitochondrial complex inhibitors, or ER stress inducers, and the cells were incubated at 37 °C. Fluorescence intensity was recorded every 10 min for 200 min at an excitation wavelength of 500 nm and an emission wavelength of 650 nm using a microplate reader. The data are presented as ratios relative to the control and shown as the mean ± SEM from three independent experiments.

### ELISA for sFlt1 and PlGF

Conditioned culture media were centrifuged at 10 000 ***g*** for 10 min at 4 °C to remove cellular debris. The concentrations of soluble fms‐like tyrosine kinase‐1 (sFlt1) and placental growth factor (PlGF) in the supernatants were determined using a Human VEGF R1/Flt‐1 Immunoassay Kit (R&D Systems, Minneapolis, MN, USA) and a Human PlGF ELISA Kit (Abcam, Tokyo, Japan), respectively, according to the manufacturers' instructions. Optical density was measured at 450 nm using a microplate reader. Analyte concentrations were calculated from standard curves, and the data are presented as the mean ± SEM from three independent experiments [18].

### Intracellular reactive oxygen species (ROS) assay

Intracellular reactive oxygen species (ROS) levels were measured using a highly sensitive 2′,7′‐dichlorodihydrofluorescein diacetate (DCFH‐DA)–based ROS detection kit (Dojindo) according to the manufacturer's instructions. TSCs were seeded into 96‐well plates and cultured under syncytiotrophoblast differentiation conditions for 48 h at 37 °C. The supernatant was then removed, and the cells were incubated with the DCFH‐DA working solution at 37 °C for 30 min. After incubation, the cells were gently washed with assay buffer, and changes in ROS levels were assessed. Fluorescence intensity was quantified using a microplate reader at excitation/emission wavelengths of 490/540 nm. The data are presented as ratios of the control and shown as mean ± SEM from three independent experiments [9].

### Western blotting

Conditioned media were centrifuged to remove debris, and equal volumes of the supernatants were subjected to SDS/PAGE. After blocking with Bullet Blocking One (Nacalai Tesque, Kyoto, Japan), the membranes were incubated with a primary antibody against hCG beta (1 : 5000, BC022796; ProteinTech, Chicago, IL, USA). Immunoreactive bands were detected using enhanced chemiluminescence (Merck Millipore, Burlington, MA, USA) after incubation with HRP‐conjugated secondary antibodies, and signals were visualized using a C‐DiGit Blot Scanner (LI‐COR) [19].

### Transfection of small interfering (si)RNA

Trophoblast stem cells (TSCs) were transfected with nontargeting control and DHODH siRNAs (each 50 nm; Sigma‐Aldrich) using Lipofectamine RNAiMAX (Thermo Fisher Scientific) according to the manufacturer's instructions. Cells were collected 48 h after transfection for subsequent analyses.

### Statistical analysis

The data are presented as the mean ± standard errors of the mean (SEMs). Statistical comparisons were performed using Tukey's test. A *P* value < 0.05 was considered to indicate statistical significance. All analyses were conducted using the R software (version 4.0.5; https://www.r‐project.org).

For RNA‐seq analyses, differential expression was considered statistically significant if the false discovery rate (FDR)‐adjusted *P* value (*q* value) was < 0.05, as specified in the text.

## Results

### DHODH inhibition impairs syncytialization in TSCs

**Fig. 1:**
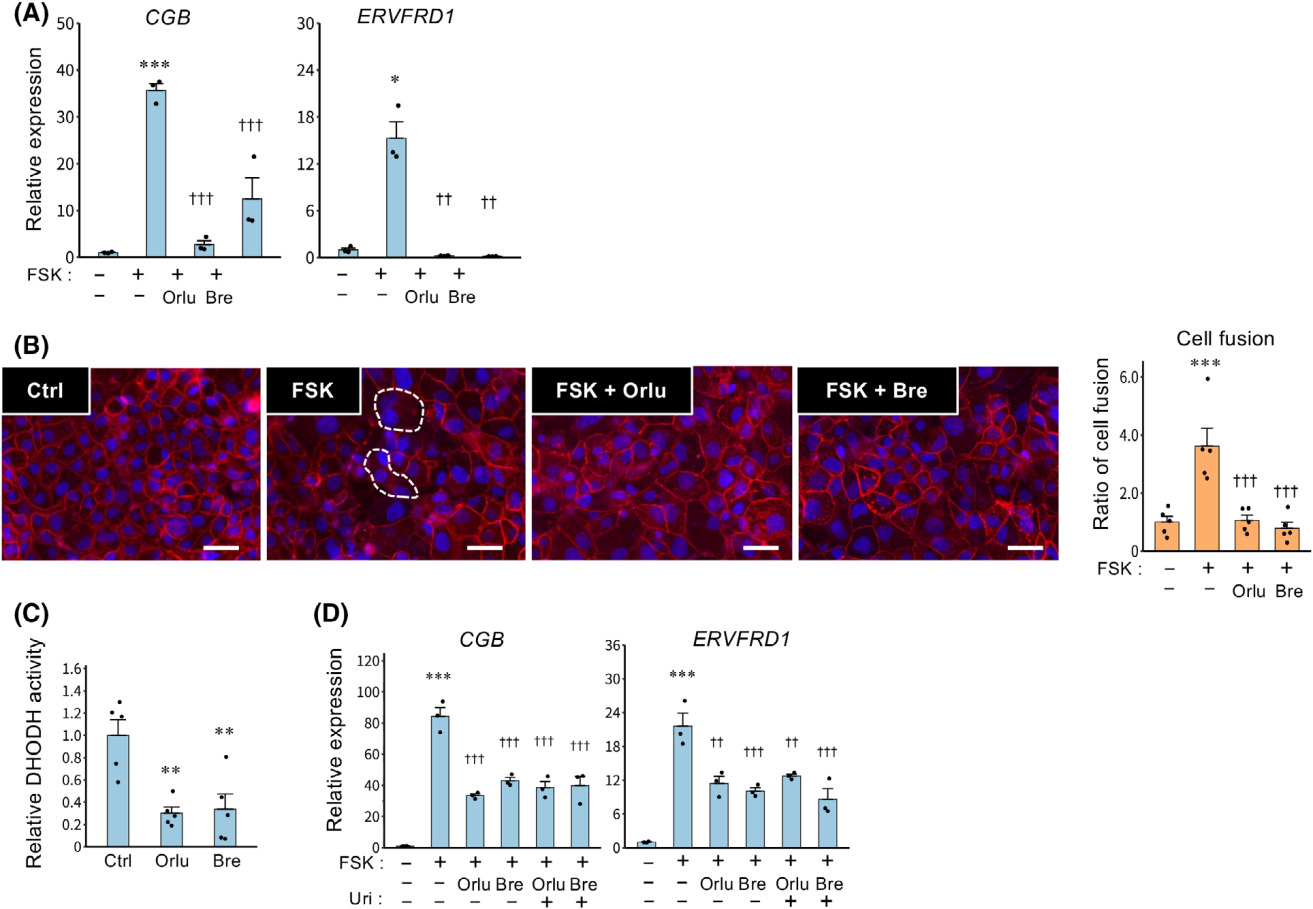
Effects of DHODH inhibitors on FSK‐induced syncytialization in TSCs. (A, B) Human trophoblast stem cells (TSCs) were treated with orludostat (Orlu, 1 nm) or brequinar (Bre, 25 nm) during differentiation induced by forskolin (FSK, 2.5 μm) for 48 h. (A) Expression of syncytialization markers *CGB* and *ERVFRD1* mRNAs is presented as the mean ± SEM from three independent experiments. *P < 0.05, ***P < 0.001 vs. Ctrl; ^††^ *P* < 0.01, ^†††^ *P* < 0.001 vs. FSK (Tukey's test). (B) Cells were stained with anti‐E‐cadherin antibody (red, plasma membrane) and DAPI (blue, nuclei) to visualize syncytialization. A representative image from three independent experiments is shown; syncytialized cells are indicated by dashed outlines (left panel). Scale bar = 100 μm. The number of syncytialized cells was quantified in five randomly selected fields per experiment (right panel). The data are presented as ratios to the control and are expressed as the mean ± SEM. ***P < 0.001 vs. Ctrl; ^†††^ *P* < 0.001 vs. FSK (Tukey's test). (C) DHODH enzymatic activity was assessed in TSC lysates after treatment with Orlu (1 nm) or Bre (25 nm) for 1 h. Enzyme activity is presented as ratios to the control and are expressed as the mean ± SEM. **P < 0.01 vs. Ctrl (Tukey's test). (D) TSCs were treated with Orlu (1 nm) or Bre (25 nm) in the presence or absence of uridine (Uri, 100 μm) during differentiation induced by FSK (2.5 μm) for 48 h. ***P < 0.001 vs. Ctrl; ^††^ *P* < 0.01, ^†††^ *P* < 0.001 vs. FSK (Tukey's test).

To investigate whether DHODH is involved in syncytialization, TSCs were treated with DHODH inhibitors Orlu or Bre, which bind to the ubiquinone‐binding pocket of DHODH and prevent the oxidation of dihydroorotate to orotate, thereby blocking DHODH‐dependent electron transfer in the presence of differentiation inducer FSK. Treatment with Orlu or Bre decreased the expression of syncytialization markers *CGB* and *ERVFRD1* (Fig. 1A). In addition, the cell fusion assay revealed that both inhibitors reduced the number of syncytialized cells (Fig. 1B). DHODH enzymatic activity was reduced by Orlu and Bre (Fig. 1C). To examine whether suppression of *CGB* and *ERVFRD1* by DHODH inhibition is mediated by reduced levels of uridine, the final product of the pyrimidine synthesis pathway, cultures were supplemented with uridine. However, uridine supplementation failed to restore their expression levels (Fig. 1D).

### DHODH inhibition induces cellular senescence and organelle stress in TSCs

**Fig. 2:**
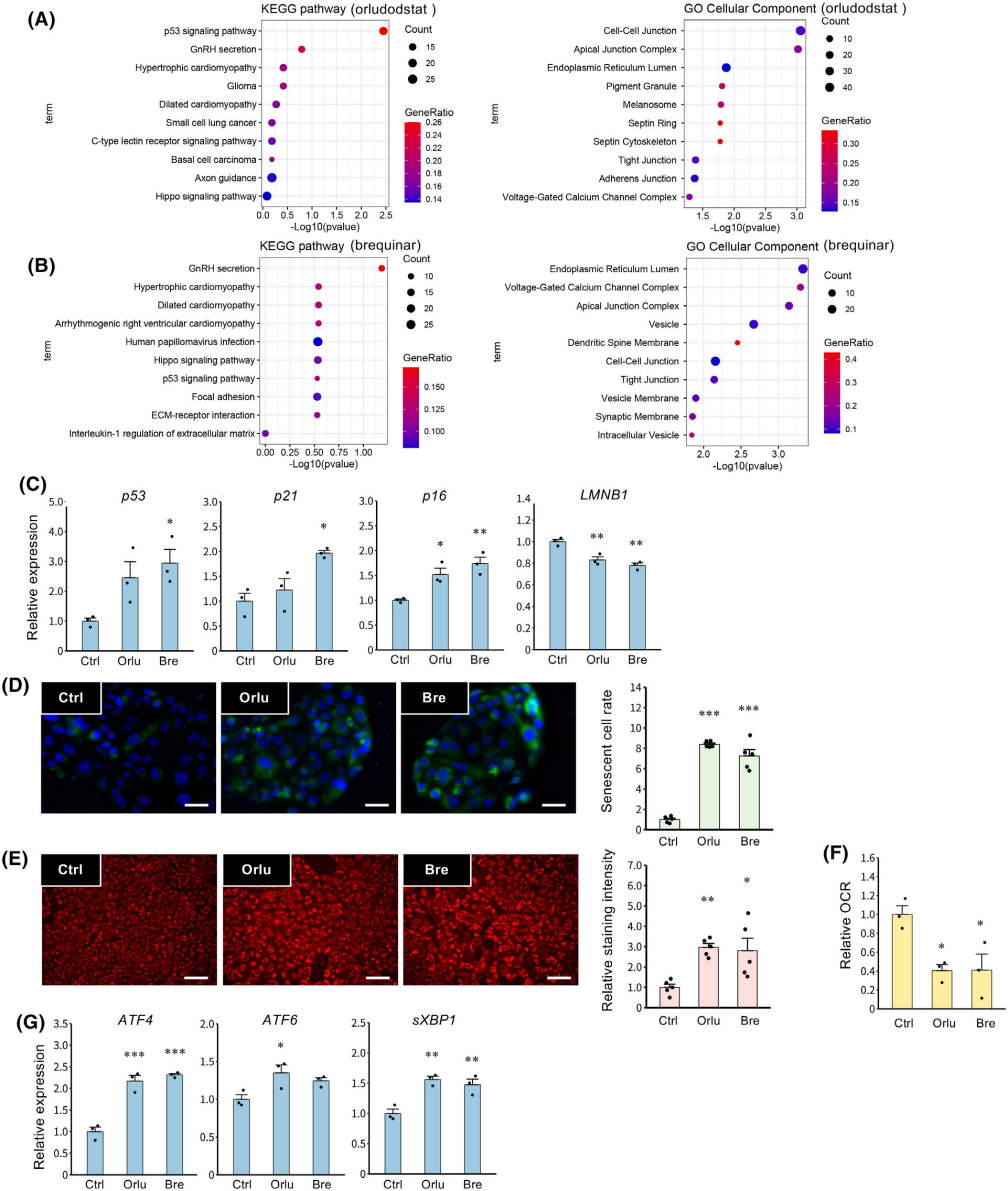
Effects of DHODH inhibitors on cellular senescence and organelle stress in TSCs. Total RNAs extracted from cells treated with FSK (2.5 μm) with or without Orlu (A; 1 nm) or Bre (B; 25 nm) for 48 h were subjected to RNA‐seq. Enrichment analyses were performed to functionally classify these genes using the database of KEGG pathways and the GO cellular components. (C) Expression of *p53, p21, p16*, and *LMNB1* mRNAs is presented as the mean ± SEM from three independent experiments. *P < 0.05, **P < 0.01 vs. Ctrl (Tukey's test). (D) Senescence‐associated β‐Gal staining was assessed. Nuclei were stained with DAPI (blue), and senescent cells were stained green. Scale bars = 20 μm. Representative data from three independent experiments are shown. The graph shows levels of the number of staining cells from three independent experiments. Values represent mean ± SEM. ***P < 0.001 vs. Ctrl (Tukey's test). (E) Mitochondrial membrane potential was assessed. A representative picture from three independent experiments is shown. Scale bar = 200 μm. The graph shows levels of membrane potential staining intensity from three independent experiments. Values represent mean ± SEM. *P < 0.05, **P < 0.01 vs. Ctrl (Tukey's test). (F) Oxygen consumption rate (OCR) was assessed. Data are presented as ratios relative to the control and are expressed as the mean ± SEM from three independent experiments. *P < 0.05 vs. Ctrl (Tukey's test). (G) Expression of *ATF4*, *ATF6*, and *sXBP1* mRNAs is presented as the mean ± SEM from three independent experiments. *P < 0.05, **P < 0.01, ***P < 0.001 vs. Ctrl (Tukey's test).

To identify DEGs induced by DHODH inhibition, RNA‐seq analysis was performed using FSK‐stimulated cells treated with Orlu (Fig. 2A) or Bre (Fig. 2B). Enrichment analysis of the upregulated expressed genes using the KEGG pathway and GO databases revealed a significant association with the p53 signaling pathway and ER‐related genes (Fig. 2A,B). Based on the enrichment analyses, the effects of DHODH inhibitors on cellular senescence and organelle stress were evaluated. Treatment with Bre increased the expression of the senescence markers *p53*, *p21*, and *p16* while decreasing *LMNB1* expression. Orlu also increased *p16* and decreased *LMNB1* expression (Fig. 2C). Furthermore, both inhibitors increased the number of SA‐β‐Gal‐positive senescent cells (Fig. 2D). Meanwhile, treatment with Orlu and Bre not only increased the mitochondrial membrane potential (Fig. 2E) but also decreased the oxygen consumption rate (OCR), an indicator of mitochondrial respiratory activity (Fig. 2F). In addition, Orlu and Bre upregulated the expression of ER stress markers, including *ATF4*, *ATF6*, and *sXBP1* (Fig. 2G). To confirm that these effects were specifically attributable to DHODH inhibition rather than off‐target actions of the chemical inhibitors, DHODH expression was knocked down in TSCs (Fig. S1A). Consistent with pharmacological inhibition, DHODH knockdown suppressed cell fusion (Fig. S1B) and increased the number of SA‐β‐Gal‐positive senescent cells (Fig. S1C). DHODH knockdown also upregulated the expression of ER stress markers (Fig. S1D).

### Mitochondrial dysfunction inhibits syncytialization and induces cellular senescence in TSCs

**Fig. 3:**
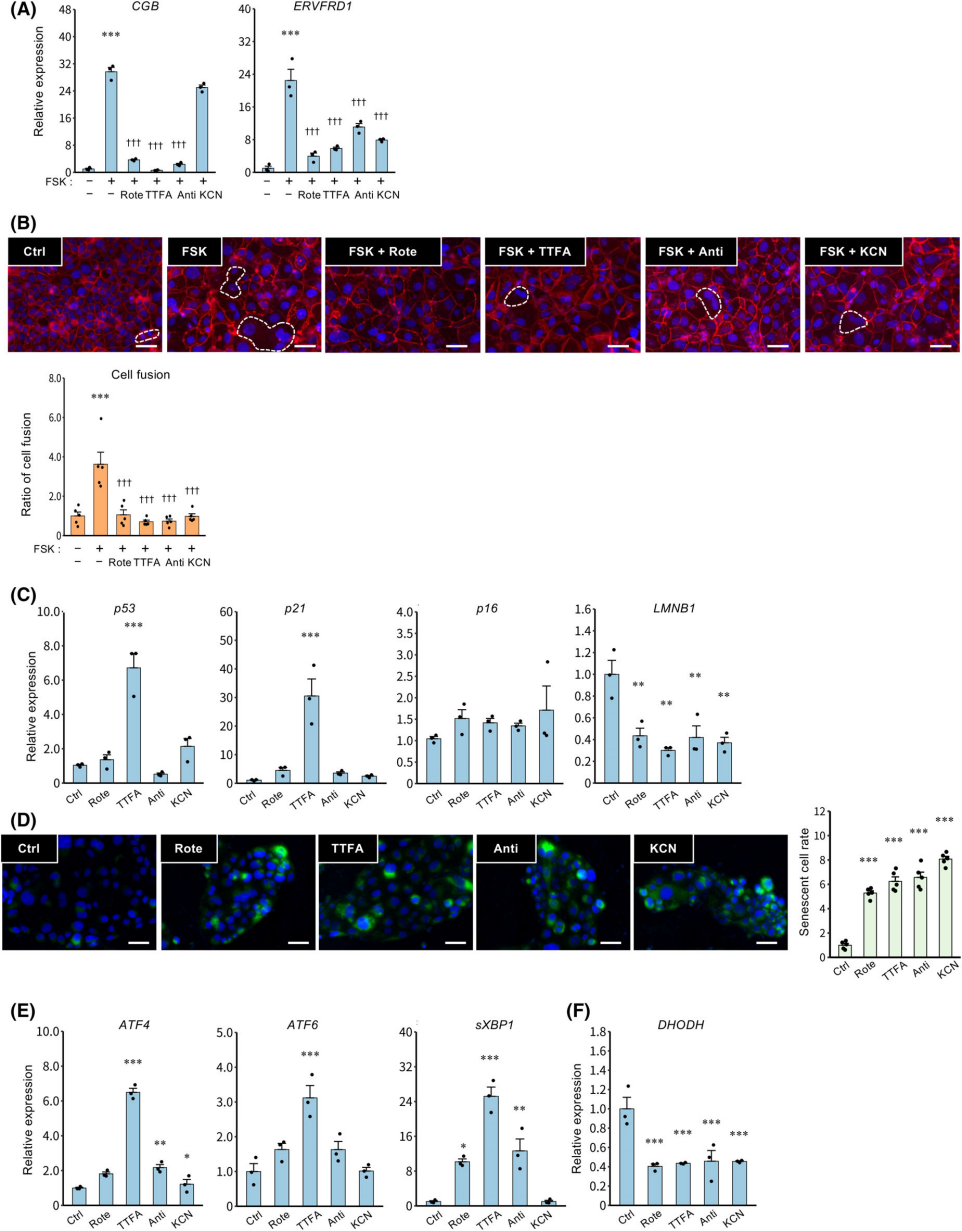
Effects of mitochondrial complex inhibitors on syncytialization and cellular senescence in TSCs. Cells were treated with rotenone (Rote 50 nm), 2‐thenoyltrifluoroacetone (TTFA, 500 μm), antimycin A (Anti, 50 nm), and potassium cyanide (KCN, 500 μm) in the presence of FSK (2.5 μm) for 48 h. (A) Expression of *CGB* and *ERVFRD1* mRNAs is presented as the mean ± SEM from three independent experiments. ***P < 0.001 vs. Ctrl; ^†††^ *P* < 0.001 vs. FSK (Tukey's test). (B) Cells were stained with anti‐E‐cadherin antibody (red, plasma membrane) and DAPI (blue, nuclei) to visualize syncytialization. A representative image from three independent experiments is shown; syncytialized cells are indicated by dashed outlines (upper panel). Scale bar = 100 μm. The number of syncytialized cells was quantified in five randomly selected fields per experiment (lower panel). The data are presented as ratios to the control and are expressed as the mean ± SEM. ***P < 0.001 vs. Ctrl; ^†††^ *P* < 0.001 vs. FSK (Tukey's test). (C–F) Cells were treated with Rote (50 nm), TTFA (500 μm), Anti (50 nm) and KCN (500 μm) for 48 h. (C) Expression of *p53, p21, p16* and *LMNB1* mRNAs is presented as the mean ± SEM from three independent experiments. **P < 0.01, ***P < 0.001 vs. Ctrl (Tukey's test). (D) Senescence‐associated β‐Gal staining was assessed. Nuclei were stained with DAPI (blue), and senescent cells were stained green. Scale bars = 20 μm. Representative data from three independent experiments are shown. The graph shows levels of the number of staining cells from three independent experiments. Values represent mean ± SEM. ***P < 0.001 vs. Ctrl (Tukey's test). (E) mRNA expression of *ATF4*, *ATF6*, and *sXBP1* mRNAs is presented as the mean ± SEM from three independent experiments. *P < 0.05, **P < 0.01, ***P < 0.001 vs. Ctrl (Tukey's test). (F) Expression of *DHODH* mRNA is presented as the mean ± SEM from three independent experiments. ***P < 0.001 vs. Ctrl (Tukey's test).

To investigate how DHODH inhibition contributes to mitochondrial dysfunction, TSCs were treated with inhibitors of mitochondrial respiratory complexes I‐IV: Rote, TTFA, Anti, or KCN. Upregulation of *CGB* expression induced by FSK was suppressed by Rote, TTFA, and Anti, while *ERVFRD1* expression was suppressed by all inhibitors (Fig. 3A). In addition, all inhibitors reduced the number of syncytialized cells (Fig. 3B). Although *p53* and *p21* expression were increased only by TTFA (Fig. 3C), reduced *LMNB1* expression and the increase in the number of SA‐β‐Gal–positive senescent cells were observed with all inhibitors (Fig. 3D). Furthermore, treatment with TTFA upregulated the expression of *ATF4*, *ATF6*, and *sXBP1* (Fig. 3E). *DHODH* expression was suppressed by all inhibitors (Fig. 3F).

### ER stress inhibits syncytialization and induces cellular senescence in TSCs

**Fig. 4:**
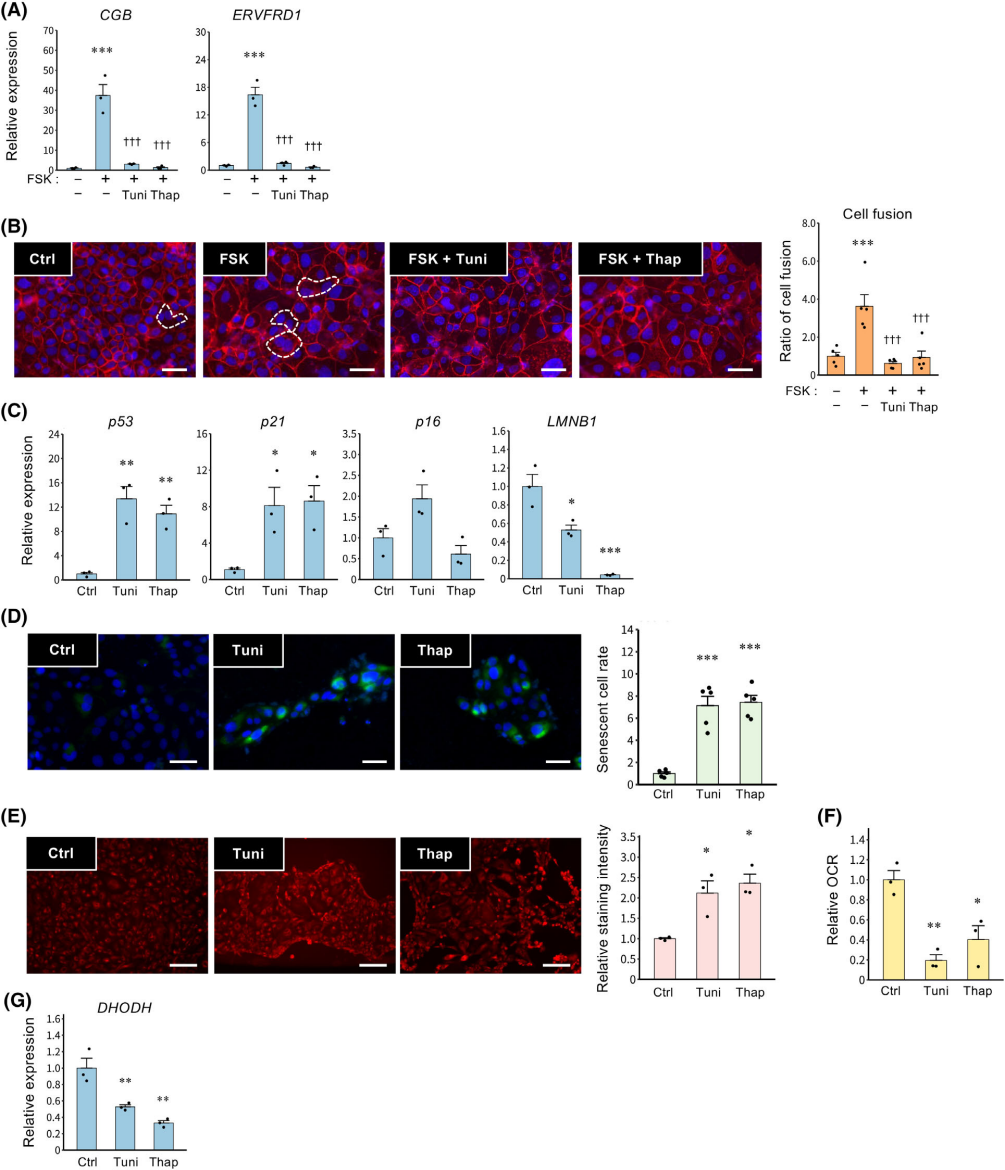
Effects of ER stress inducers on syncytialization and cellular senescence in TSCs. Cells were treated with tunicamycin (Tuni, 1 μm) or thapsigargin (Thap, 50 nm) in the presence of FSK (2.5 μm) for 48 h. (A) Expression of *CGB* and *ERVFRD1* mRNAs is presented as the mean ± SEM from three independent experiments. ***P < 0.001 vs. Ctrl; ^†††^ *P* < 0.001 vs. FSK (Tukey's test). (B) Cells were stained with anti‐E‐cadherin antibody (red, plasma membrane) and DAPI (blue, nuclei) to visualize syncytialization. A representative image from three independent experiments is shown; syncytialized cells are indicated by dashed outlines. Scale bar = 100 μm. The number of syncytialized cells was quantified in five randomly selected fields per experiment. The data are presented as ratios to the control and are expressed as the mean ± SEM. ***P < 0.001 vs. Ctrl; ^†††^ *P* < 0.001 vs. FSK (Tukey's test). (C–F) Cells treated with Tuni (1 μm) or Thap (50 nm) for 48 h. (C) Expression of *p53*, *p21*, *p16*, and *LMNB1* mRNAs is presented as the mean ± SEM from three independent experiments. *P < 0.05, **P < 0.01, ***P < 0.001 vs. Ctrl (Tukey's test). (D) Senescence‐associated β‐Gal staining was assessed. Nuclei were stained with DAPI (blue), and senescent cells were stained green. Scale bars = 20 μm. Representative data from three independent experiments are shown. The graph shows levels of the number of staining cells from three independent experiments. Values represent mean ± SEM. ***P < 0.001 vs. Ctrl (Tukey's test). (E) Mitochondrial membrane potential was assessed. A representative picture from three independent experiments is shown. Scale bar = 200 μm. The graph shows levels of membrane potential staining intensity from three independent experiments. Values represent mean ± SEM. *P < 0.05 vs. Ctrl (Tukey's test). (F) OCR was assessed. Data are presented as ratios relative to the control and are expressed as the mean ± SEM from three independent experiments. *P < 0.05, **P < 0.01 vs. Ctrl (Tukey's test). (G) Expression of *DHODH* mRNA is presented as the mean ± SEM from three independent experiments. **P < 0.01 vs. Ctrl (Tukey's test).

Since inhibition of DHODH and mitochondrial complexes upregulated the expression of ER stress‐related genes, the effects of ER stress on syncytialization and cellular senescence were evaluated. Treatment with ER stress inducers Tuni and Thap suppressed the expression of *CGB* and *ERVFRD1* (Fig. 4A) and inhibited cell fusion (Fig. 4B). Tuni and Thap elevated the expression of *p53* and *p21*, while decreasing *LMNB1* (Fig. 4C). Furthermore, the inducers increased the number of SA‐β‐Gal‐positive senescent cells (Fig. 4D). Both inducers also elevated mitochondrial membrane potential (Fig. 4E) and decreased the OCR (Fig. 4F). In addition, ER stress inducers suppressed *DHODH* expression (Fig. 4G).

### DHODH inhibition and organelle stress alters the expression and secretion of HDP‐related genes

**Fig. 5:**
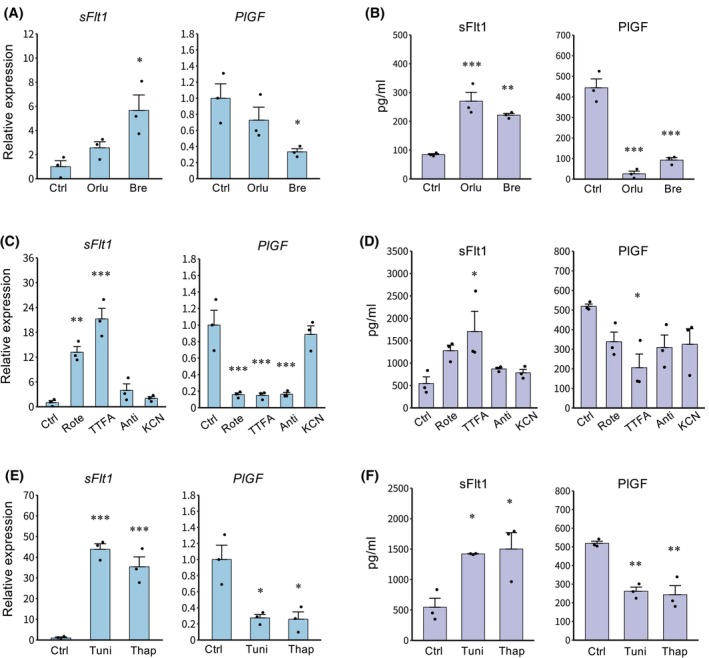
Effects of DHODH inhibition and organelle stress on the expression and secretion of HDP‐related gene markers in TSCs. (A, B) Cells were treated with Orlu (1 nm) or Bre (25 nm) for 48 h. (A) Expression of *sFlt1* and *PlGF* mRNAs is presented as the mean ± SEM from three independent experiments. *P < 0.05, vs. Ctrl (Tukey's test). (B) Secreted sFlt1 and PlGF levels are presented as the mean ± SEM from three independent experiments. **P < 0.01, ***P < 0.001 vs. Ctrl (Tukey's test). (C, D) Cells were treated with Rote (50 nm), TTFA (500 μm), Anti (50 nm), and KCN (500 μm) for 48 h. (C) Expression of *sFlt1* and *PlGF* mRNAs is presented as the mean ± SEM from three independent experiments. **P < 0.01, ***P < 0.001 vs. Ctrl (Tukey's test). (D) Secreted sFlt1 and PlGF levels are presented as the mean ± SEM from three independent experiments. *P < 0.05 vs. Ctrl (Tukey's test). (E, F) Cells were treated with Tuni (1 μm) and Thap (50 nm) for 48 h. (E) Expression of *sFlt1* and *PlGF* mRNAs is presented as the mean ± SEM from three independent experiments. *P < 0.05, ***P < 0.001 vs. Ctrl (Tukey's test). (F) Secreted sFlt1 and PlGF levels are presented as the mean ± SEM from three independent experiments. *P < 0.05, **P < 0.01 vs. Ctrl (Tukey's test).

To examine whether DHODH inhibition and organelle stress contribute to the pathophysiology of HDP, we assessed the expression and secretion of HDP severity markers sFlt1 and PlGF. Treatment with Bre increased *sFlt1* and decreased *PlGF* mRNA expression (Fig. 5A), and both Orlu and Bre increased the secretion of sFlt1 and decreased PlGF (Fig. 5B). Similarly, the mitochondrial complex I and II inhibitors Rote and TTFA increased *sFlt1* and decreased *PlGF* mRNA expression (Fig. 5C), and TTFA treatment increased sFlt1 and decreased PlGF secretion (Fig. 5D). The ER stress inducers Tuni and Thap also increased *sFlt1* and decreased *PlGF* mRNA expression (Fig. 5E), and both inducers increased sFlt1 secretion while reducing PlGF secretion (Fig. 5F).

### Quercetin and riboflavin restore syncytialization‐ and HDP‐related marker expression

**Fig. 6:**
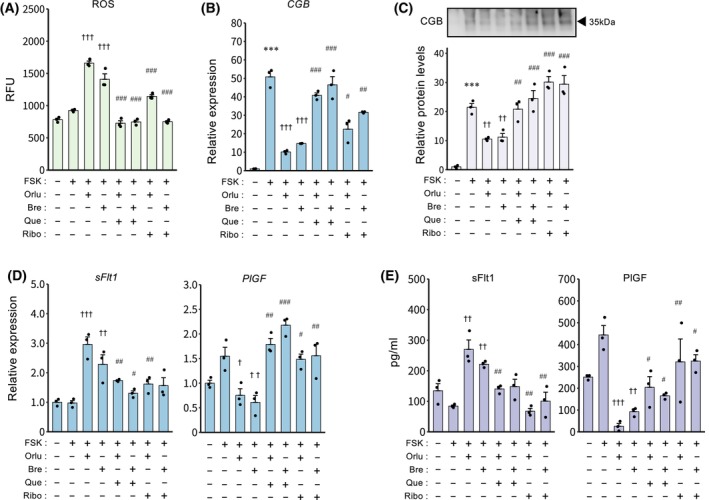
Effects of quercetin and riboflavin on syncytialization and the expression of HDP‐related markers in TSCs. Cells were treated with Orlu (1 nm) or Bre (25 nm), and/or Que (5 μm) or Ribo (25 μm) in the presence of FSK (2.5 μm) for 48 h. (A) Intracellular ROS levels are presented as the mean ± SEM from three independent experiments. ^†††^ *P* < 0.001 vs. FSK; ^###^ *P* < 0.001 vs. FSK/Orlu or FSK/Bre (Tukey's test). (B) Expression of *CGB* mRNA is presented as the mean ± SEM from three independent experiments. ***P < 0.001 vs. Ctrl; ^†††^ *P* < 0.001 vs. FSK; ^#^ *P* < 0.05, ^##^ *P* < 0.01, ^###^ *P* < 0.001 vs. FSK/Orlu or FSK/Bre (Tukey's test). (C) Immunoblotting for hCGB in conditioned medium. Representative data from three independent experiments are shown. The graph shows hCGB band intensities quantified from three independent experiments and normalized to the Ctrl. ***P < 0.001 vs. Ctrl; ^††^ *P* < 0.01 vs. FSK; ^##^ *P* < 0.01, ^###^ *P* < 0.001 vs. FSK/Orlu or FSK/Bre (Tukey's test). (D) Expression of *sFlt1* and *PlGF* mRNAs is presented as the mean ± SEM from three independent experiments. ^††^ *P* < 0.01, ^†††^ *P* < 0.001 vs. FSK; ^#^ *P* < 0.05, ^##^ *P* < 0.01, ^###^ *P* < 0.001 vs. FSK/Orlu or FSK/Bre (Tukey's test). (E) Secreted sFlt1 and PlGF levels are presented as the mean ± SEM from three independent experiments. ^††^ *P* < 0.01, ^†††^ *P* < 0.001 vs. FSK; ^#^ *P* < 0.05, ^##^ *P* < 0.01, vs. FSK/Orlu or FSK/Bre (Tukey's test).

To examine whether mitochondrial protective compounds could restore genes related to syncytialization and HDP that were suppressed by Orlu or Bre, TSCs were treated with Que, an antioxidant, and Ribo, a cofactor that enhances mitochondrial complex I and II activity. The increase in ROS levels induced by Orlu or Bre was attenuated by treatment with Que or Ribo (Fig. 6A). Furthermore, either Que or Ribo restored CGB mRNA expression and secreted protein levels that had been reduced by DHODH inhibition (Fig. 6B,C). Que or Ribo treatment also reversed the expression of *sFlt1* and *PlGF* (Fig. 6D). Similarly, the Orlu‐ and Bre‐induced increase in sFlt1 secretion was mitigated by Que and Ribo, and the decrease in PlGF secretion by the inhibitors was reversed by the same treatments (Fig. 6E).

## Discussion

**Fig. 7:**
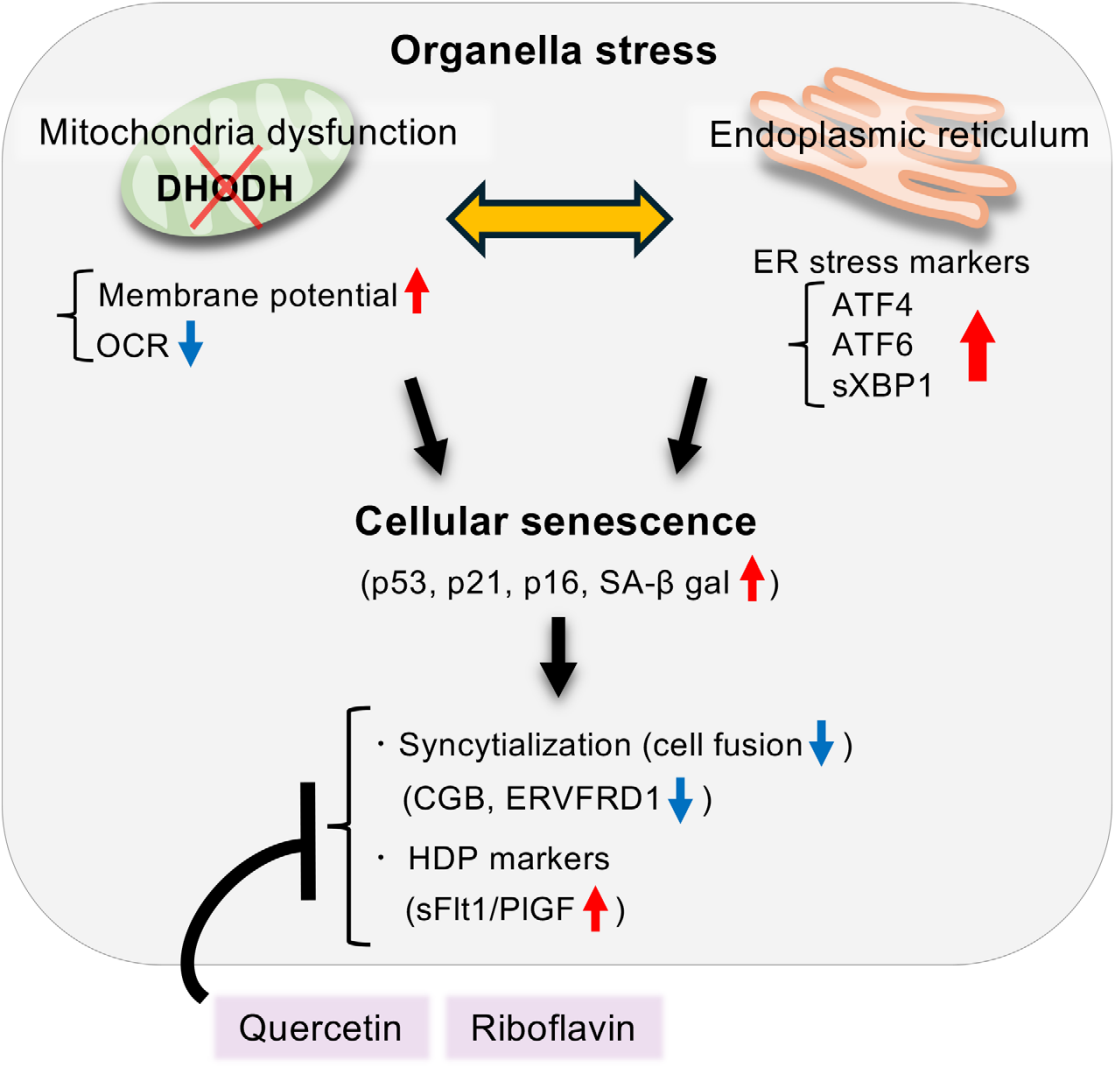
The schematic diagram illustrating the mechanism by which dihydroorotate dehydrogenase (DHODH) regulates trophoblast differentiation and contributes to HDPs. Reduced expression of mitochondrial DHODH was observed in early‐onset HDP placentas. DHODH inhibition impairs syncytialization, the fusion of cytotrophoblasts into syncytiotrophoblasts, in human trophoblast stem cells (TSCs). This impairment is mediated by the induction of organelle stress, specifically mitochondrial dysfunction (reduced oxygen consumption rate [OCR] and increased mitochondrial membrane potential) and endoplasmic reticulum (ER) stress (evidenced by upregulated markers: ATF4, ATF6, and sXBP1). These organelle stresses, in turn, induce cellular senescence. Both DHODH inhibition and organelle stresses may operate through a bidirectional positive feedback mechanism. Functionally, this dysfunction elevates the levels of HDP severity markers, characterized by increased sFlt1 and decreased PlGF expression and secretion, reflecting placental dysfunction. Treatment with mitochondrial activators such as quercetin (Que) and riboflavin (Ribo) partially restores CGB expression (a syncytialization marker) and normalizes the altered sFlt1 and PlGF levels.

In this study, we demonstrate that DHODH inhibition induces cellular senescence through mitochondrial dysfunction and ER stress, leading to impaired syncytialization in TSCs. These dysfunctions may exacerbate HDPs (Fig. 7).

The DHODH inhibitors Orlu and Bre not only inhibited syncytialization in TSCs but also induced upregulation of senescence‐associated genes and accumulation of senescent cells. RNA‐seq analysis revealed that DHODH inhibition was associated with genes related to the p53 signaling pathway. DHODH is a rate‐limiting enzyme in the *de novo* pyrimidine synthesis pathway, and its inhibition may lead to pyrimidine depletion, resulting in the activation of DNA damage responses [20, 21]. To assess whether DHODH‐induced syncytialization defects stemmed from impaired pyrimidine synthesis, we supplemented the cultures with uridine, the final product of this pathway. However, uridine supplementation did not restore cell fusion in DHODH‐inhibited trophoblast cells. This indicates that pharmacological inhibition of DHODH with Orlu or Bre suppresses syncytialization independently of the *de novo* pyrimidine synthesis pathway. In addition to cellular senescence, DHODH inhibition induced mitochondrial dysfunction, as evidenced by increased mitochondrial membrane potential and markedly reduced OCR. Furthermore, the elevated expression of ER stress markers suggests an association between DHODH inhibition and organelle stress. Similar to DHODH inhibition, inhibition of mitochondrial respiratory complexes increased cellular senescence and induced ER stress, especially complex II. Considering that DHODH physically associates with mitochondrial complexes II and III [22], it is likely that mitochondrial complex II serves as a key mediator of DHODH‐induced cellular senescence and ER stress. Mitochondrial dysfunction and ER stress are known to be closely interconnected through ER–mitochondria communication [23]. Because DHODH depends on electron transfer to ubiquinone within the mitochondrial inner membrane, its activity and expression have been reported to be dependent on the integrity of the electron transport chain [24]. Thus, the changes in DHODH expression observed after treatment with mitochondrial inhibitors or ER‐stress inducers may reflect stress responses arising from disrupted mitochondrial function. However, the precise mechanisms regulating DHODH expression under these stress conditions remain unclear and warrant further investigation. Regarding the expression of ER stress‐related genes, DHODH inhibition and mitochondrial complex II inhibition were associated with altered expression of unfolded protein response (UPR) transcription factors, including ATF4, ATF6, and sXBP1. Further, chronic activation of the UPR has been reported to promote p53 signaling and senescence‐associated gene expression [25]. Leflunomide, an approved DHODH inhibitor, has been reported to trigger ER stress in hepatocytes, which can be attenuated by JNK inhibition [26], suggesting that comparable signaling cascades may be involved in cellular senescence in trophoblasts. Collectively, these findings suggest that DHODH inhibition, mitochondrial complex II dysfunction, and ER stress are closely interconnected and may contribute to cellular senescence and impaired syncytialization.

Pregnancy is characterized by dramatic alterations in hormonal balance and energy metabolism to nurture and support the developing fetus [27]. However, disruption of these systems may alter the stress responsiveness of trophoblasts and consequently lead to organelle dysfunction [28, 29]. Although the specific form of organelle stress that serves as the initial trigger remains unclear, pregnancy‐associated changes may sensitize trophoblasts to organelle stress, which can in turn activate the p53 pathway through mitochondrial dysfunction and ER stress, thereby establishing a feedback loop that promotes cellular senescence. Importantly, chronic exposure to intracellular organelle stress in the placenta has been reported to contribute to the development of HDPs. Furthermore, senescence‐associated secretory phenotype and mitochondrial dysfunction have been detected in placentas from HDP patients, and these features are considered key components of disease progression [30, 31, 32]. Moreover, advanced maternal age has been associated with decreased placental sirtuin 1 level, enhanced oxidative stress, and vascular malperfusion, highlighting that advanced maternal age may exacerbate placental organelle stress [33]. Supporting this concept, we found that treatment with Bre, Rote, TTFA, Tuni, and Thap increased *sFlt1* secretion and decreased *PlGF* secretion, which is consistent with features observed in HDPs.

Both Que and Ribo restored *CGB* expression suppressed by DHODH inhibition and normalized the altered expression of *sFlt1* and *PlGF*. This reveals that DHODH inhibition‐induced trophoblast dysfunctions are mediated through mitochondrial impairment. Que exerts a wide range of biological effects, including the prevention of excessive ROS accumulation, stabilization of mitochondrial function, and modulation of inflammatory signaling [34]. Consistent with previous findings, Que and Ribo suppressed the excessive ROS induced by DHODH inhibition in this study. By contrast, Ribo functions as an essential cofactor for flavoproteins, playing a critical role in the activity of mitochondrial complex II. In addition, the riboflavin transporter SLC52A1 has been reported to suppress cellular senescence by inhibiting the AMPK‐p53 pathway through Ribo uptake [35]. Considering that DHODH physically associates with complex II, the restorative effect of Ribo observed in this study likely reflects the enhancement of complex II function. Taken together, these findings suggest that the DHODH–mitochondrial complex II axis may represent a promising therapeutic target for mitigating organelle stress in HDPs.

In conclusion, DHODH inhibition and organelle stresses, including mitochondrial complex II inhibition and endoplasmic reticulum stress, may operate through a bidirectional positive feedback mechanism, leading to suppression of syncytialization and induction of cellular senescence in human trophoblast stem cells. Thus, targeting the DHODH‐mitochondrial axis may offer novel therapeutic avenues for HDP intervention.

## Conflict of interest

The authors declare no conflict of interest.

## Author contributions

K.Y., A.H., and Y.K. performed experiments and analyses. K.Y., K.K., and K.T. wrote the main manuscript text and K.Y., K.K., and A.H. prepared all figures and tables. K.Y., K.K., A.T., M.Y., and K.T. were involved in the planning of the entire experimentation. All authors reviewed the manuscript.

## Supporting information


**Fig. S1.** DHODH knockdown suppresses syncytialization and induces cellular senescence.
**Fig. S2.** Full‐length western blot images corresponding to Fig 6B.

**Table S1.** Primers for real‐time PCR analyses.

## Data Availability

The data that support the findings of this study are available from the corresponding author upon reasonable request.
