# Supplementary material for: Dihydroorotate dehydrogenase (DHODH) regulates trophoblast syncytialization through organelle stress–induced cellular senescence
Source: FEBS Open Bio. 2026 Jan 16;16(6):1166–80. doi: 10.1002/2211-5463.70194 (PMC13238865; doi:10.1002/2211-5463.70194)

Supplementary Figure

Figure S1. DHODH knockdown suppresses syncytialization and induces cellular senescence

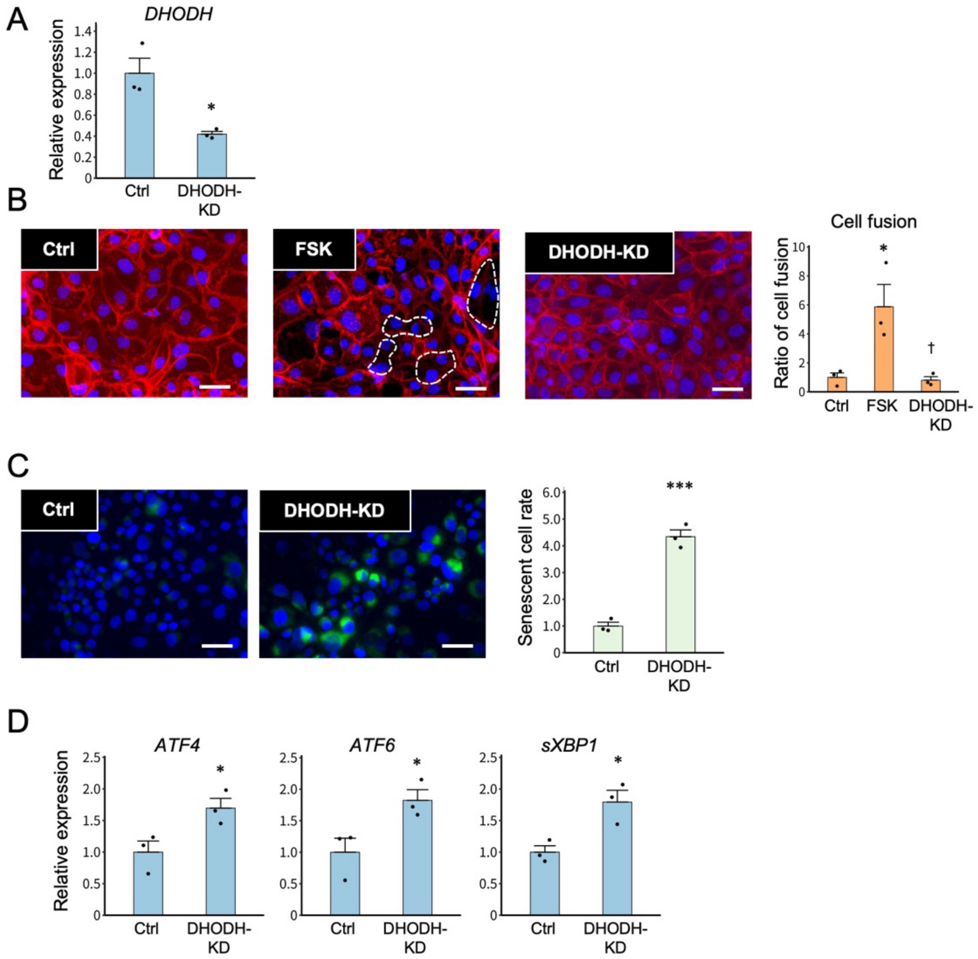

Figure S1. The effects of knockdown of DHODH on syncytialization and cellular senescence.

(A) Expression of *DHODH* mRNA is presented as the means ± SEMs from three independent experiments. *P < 0.05 *vs*. Ctrl. (B) Cells were stained with anti-E-cadherin antibody (red, plasma membrane) and DAPI (blue, nuclei) to visualize syncytialization. A representative image from three independent experiments is shown; syncytialized cells are indicated by dashed outlines (left panel). Scale bar = 100 µm. The number of syncytialized cells was quantified in three randomly selected fields per experiment (right panel). The data are presented as ratios to the control and are expressed as the means ± SEMs. *P < 0.001 vs. Ctrl; †P < 0.01 *vs.* FSK. (C) Senescence-associated β-Gal staining was assessed. Nuclei were stained with DAPI (blue), and senescent cells were stained green (left panel). Scale bars = 20 µm. Representative data from three independent experiments are shown. The graph shows levels of the number of staining cells from three independent experiments. Values represent mean ± SEM. ***P < 0.001 *vs*. Ctrl. (D) Expression of ATF4, ATF6, and sXBP1 mRNAs is presented as the means ± SEMs from three independent experiments. *P < 0.05 *vs*. Ctrl.

Figure S2. Full-length Western blot images corresponding to Figures 6B.

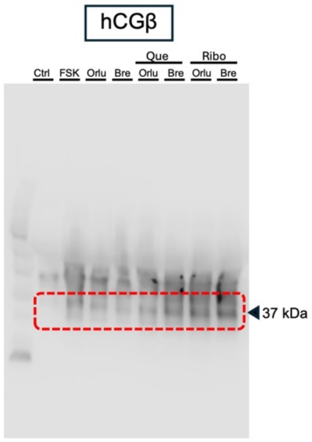

Supplement: Supplementary file 1 — Fig. S1. DHODH knockdown suppresses syncytialization and induces cellular senescence. Fig. S2. Full‐length western blot images corresponding to Fig 6B. [file FEB4-16-1166-s002.pdf]
