# Supplementary material for: Dihydroorotate dehydrogenase (DHODH) regulates trophoblast syncytialization through organelle stress–induced cellular senescence
Source: FEBS Open Bio. 2026 Jan 16;16(6):1166–80. doi: 10.1002/2211-5463.70194 (PMC13238865; doi:10.1002/2211-5463.70194)
Supplement: Supplementary file 2 — Table S1. Primers for real‐time PCR analyses. [file FEB4-16-1166-s001.pdf]

Table S1. Primers for real-time PCR analyses

Name (Accession No.)	Sequence	Product length (bp)
GAPDH NM_002046.7	F: 5'- AGCCACATCGCTCAGACA -3'<br>R: 5'- GCCCAATACGACCAAATCC -3'	66
DHODH NM_001361.5	F: 5'- GCCATAAATTCCGAAATCCAG -3'<br>R: 5'- ACAGCTTGGTCCTCAGGGAG -3'	175
CGB NM_000737.3	F: 5'- CCTGGCCTTGTCTACCTCTT -3'<br>R: 5'- GGCTTTATACCTCGGGGTTG -3'	108
ERVFRD1 NM_207582.2	F: 5'- CCAAATTCCCTCCTCTCCTC -3'<br>R: 5'- CGGGTGTTAGTTTGCTTGGT -3'	115
p53 NM_000546.6	F: 5'- TCTGACTGTACCACCATCCACTA -3'<br>R: 5'- CAAACACGCACCTCAAAGC -3'	146
CDKN1A NM_000389.5	F: 5'- GACTTTGTCACCGAGACACC -3'<br>R: 5'- GACAGGTCCACATGGTCTTC -3'	188
CDKN2A NM_000077.5	F: 5'- CCAACGCACCGAATAGTTACG -3'<br>R: 5'- CCATCATCATGACCTGGATCG -3'	51
LMNB1 NM_005573.4	F: 5'- CTCTCGTCGCATGCTGACAG -3'<br>R: 5'- TCCCTTATTTCCGCCATCTCT -3'	51
ATF4 NM_001675.4	F: 5'- GTTCTCCAGCGACAAGGCTA -3'<br>R: 5'- ATCCTGCTTGCTGTTGTTGG -3'	88
ATF6 NM_007348.4	F: 5'- CAGACAGTACCAACGCTTATGCC -3'<br>R: 5'- GCAGAACTCCAGGTGCTTGAAG -3'	133
Spliced XBP1 NM_001079539.2	F: 5'- CTGAGTCCGAATCAGGTGCAG -3'<br>R: 5'- ATCCATGGGGAGATGTTCTGG -3'	59
sFlt1 NM_001159920.2	F: 5'- ACAATCGAGGTGAGCACTGCAA -3'<br>R: 5'- TCCGAGCCTGAAAGTTAGCAA -3'	180
PlGF NM_002632.6	F: 5'- GAACGGCTCGTCAGAGGTG -3'<br>R: 5'- ACAGTTGCAGATTCTCATCGCC -3'	188
